# Neurofilament depletion improves microtubule dynamics via modulation of Stat3/stathmin signaling

**DOI:** 10.1007/s00401-016-1564-y

**Published:** 2016-03-28

**Authors:** Preeti Yadav, Bhuvaneish T. Selvaraj, Florian L. P. Bender, Marcus Behringer, Mehri Moradi, Rajeeve Sivadasan, Benjamin Dombert, Robert Blum, Esther Asan, Markus Sauer, Jean-Pierre Julien, Michael Sendtner

**Affiliations:** Institute of Clinical Neurobiology, University of Wuerzburg, 97078 Würzburg, Germany; Institute of Anatomy and Cell Biology, University of Wuerzburg, 97070 Würzburg, Germany; Department of Psychiatry and Neurosciences, Research Centre of Institut Universitaire en Santé Mentale de Québec, University Laval, Quebec, Canada; Department of Biotechnology and Biophysics, University of Wuerzburg, 97074 Würzburg, Germany; MRC Centre for Regenerative Medicine, University of Edinburgh, Edinburgh, UK

**Keywords:** Axon degeneration, Neurofilament, Microtubules, Stathmin, Stat3

## Abstract

In neurons, microtubules form a dense array within axons, and the stability and function of this microtubule network is modulated by neurofilaments. Accumulation of neurofilaments has been observed in several forms of neurodegenerative diseases, but the mechanisms how elevated neurofilament levels destabilize axons are unknown so far. Here, we show that increased neurofilament expression in motor nerves of *pmn* mutant mice, a model of motoneuron disease, causes disturbed microtubule dynamics. The disease is caused by a point mutation in the tubulin-specific chaperone E (*Tbce*) gene, leading to an exchange of the most C-terminal amino acid tryptophan to glycine. As a consequence, the TBCE protein becomes instable which then results in destabilization of axonal microtubules and defects in axonal transport, in particular in motoneurons. Depletion of neurofilament increases the number and regrowth of microtubules in *pmn* mutant motoneurons and restores axon elongation. This effect is mediated by interaction of neurofilament with the stathmin complex. Accumulating neurofilaments associate with stathmin in axons of *pmn* mutant motoneurons. Depletion of neurofilament by *Nefl* knockout increases Stat3–stathmin interaction and stabilizes the microtubules in *pmn* mutant motoneurons. Consequently, counteracting enhanced neurofilament expression improves axonal maintenance and prolongs survival of *pmn* mutant mice. We propose that this mechanism could also be relevant for other neurodegenerative diseases in which neurofilament accumulation and loss of microtubules are prominent features.

**Electronic supplementary material:**

The online version of this article (doi:10.1007/s00401-016-1564-y) contains supplementary material, which is available to authorized users.

## Introduction

Destabilization of axon terminals and axon degeneration are key pathological features in amyotrophic lateral sclerosis (ALS) and spinal muscular atrophy (SMA), the most common forms of motoneuron disease [6, 33, 62, 68]. Despite progress in the identification of underlying gene defects [41, 58, 60], the cellular mechanisms that are responsible for loss of motor function, degeneration of neuromuscular endplates, destabilization of axons and finally death of motoneuron cell bodies are not fully understood [20]. SMA and ALS are considered both clinically and genetically as distinct disorders. However, they share common features. On the clinical side, dysfunction and degeneration of neuromuscular endplates appears as an early symptom in both disorders, and alterations of the axonal cytoskeleton are characteristic at early stages of both diseases [16, 29, 35]. Accumulation of spheroids in proximal axons is commonly observed in ALS, and these spheroids primarily contain neurofilaments [13, 23, 52]. In a mouse model of SMA, the density of intermediate filaments in proximal and distal axons is highly increased [9], similar as in tg(SOD1*G93A) mutant mice [75], a model for a common form of familial ALS. This increased density of neurofilaments coincides with lack of axonal sprouting and increased dynamics and instability of axonal microtubules [19, 22, 37, 38], indicating that the increased density of intermediate filaments and destabilization of microtubules are functionally connected.

Neurofilaments modulate microtubule stability and thus axon caliber and microtubule functions in axonal transport [49]. NFL is required for the assembly of neurofilaments, as NFM and NFH cannot form intermediate filaments in the absence of NFL [17, 18]. Abnormal accumulation of neurofilaments causes neuronal degeneration by disrupting axonal transport of cargos required for the maintenance of axon terminals [10]. Depletion of axonal neurofilaments in *Nefl*−*/*− and tg(*Prph*);*Nefl*−*/*− neurons leads to improved axonal transport of mitochondria and lysosomes [56]. It also increases life span in tg(SOD1*G85R) mutant mice [79] and attenuates the neurodegenerative disease phenotype in tau T44 transgenic mice [28]. However, the precise molecular mechanisms how neurofilament depletion stabilizes microtubules, improves axonal transport and prevents axon degeneration have remained unclear.

Here we show that NFL depletion in *pmn* mutant mice [7, 46, 66] increases microtubule stability and delays axon degeneration. Disruption of the *Nefl* gene in *pmn* mutant mice increases microtubule numbers in motor axons, and ameliorates the disease phenotype. This effect appears to be caused by interaction of NFL with a protein complex including stathmin and signal transducer and activator of transcription-3 (Stat3). Interaction of Stat3 and stathmin is increased upon NFL depletion. Enhanced Stat3–stathmin interaction inhibits stathmin action in destabilizing microtubules and increases levels of soluble tubulin heterodimers making them available for the maintenance of axonal microtubules in motoneurons. This effect could explain why neurofilament depletion delays disease onset and prolongs survival in *pmn* mutant mice, and also how increased neurofilament levels lead to axonal destabilization in a wide variety of neurodegenerative disorders.

## Materials and methods

### Description of mouse lines used in this study

Heterozygous *pmn* mutant mice originally maintained on Naval Medical Research Institute (NMRI) [64] genetic background were crossed with heterozygous *Nefl* knockout mice on a C57BL/6 genetic background [83] to produce double heterozygous *Nefl*+/−; *pmn*+/− mice. These mice were then backcrossed with NMRI genetic background for at least four generations to obtain a uniform NMRI genetic background. Subsequently, the heterozygous *Nefl*+/−; *pmn*+/− were intercrossed to obtain the mice investigated in this work. NMRI genetic background was preferred over C57BL/6 to obtain bigger litter sizes under a situation when two autosomal recessive traits (*pmn* and *Nefl*) had to be crossed to obtain the desired genotypes. *Nefl* knockout and corresponding control wild-type mice on a C57BL/6 genetic background were used for the immunoprecipitation experiments. All experimental procedures were approved by animal care and ethic committee of the University of Wuerzburg, the Veterinäramt of the City of Wuerzburg and the Regierung von Unterfranken, and were performed according to the guidelines of the European Union. All mice were maintained under a 12-h light/dark cycle with food and water ad libitum.

### Antibodies

Antibodies against neurofilament-light chain Ab9035 (Western blot), and NFL mouse monoclonal, Cat# MCA-DA2 (immunostaining), tyrosinated α-tubulin (clone YL1/2; ab6160) and stathmin-1 (clone EP1573Y; ab52630) were obtained from Abcam. The specificity of these antibodies and in particular the stathmin antibody has been tested in previous studies [5, 66]. After stathmin-1 knockout [5] or lentiviral knockdown [66] the corresponding Stathmin-1 band was completely abolished in Western blots. Neurofilament-heavy chain antibody (AB5539) was obtained from Millipore, eIF2α (D7D3), p-STAT3^Y705^ (D3A7), and Stat3 (124H6) (9139S) antibodies from Cell Signaling Technology. γ-tubulin (clone GTU-88), tau (T-6402), acetylated-α-tubulin (clone 6-11b-1; T7451) and α-tubulin (clone B-5-1-2; T5168) antibodies were purchased from Sigma-Aldrich. The Stathmin 2 (SCG10) antibody was a kind gift from the lab of Dr. Gabriele Grenningloh, EPFL [15].

### Survival of the mice

To compare survival of *pmn* mice lacking NFL, mice heterozygous for *Nefl*+/−; *pmn*+/− were intercrossed, and survival of mutant mice of either sex with different genotypes was compared. *Nefl*+*/*+;*pmn* mice were used as a control for comparison with *Nefl*+/−;*pmn* and *Nefl*−*/*−;*pmn* to analyze survival with Kaplan–Meier curves. The median survival for the mice was compared using log rank test. Mice analyzed for survival were not used for the behavior tests.

### Functional analysis of mice with rotarod and grip strength tests

Motor coordination and motor performance of the mutant mice were tested using a rotarod (Accelerating Rotarod, Ugo Basile) by recording the latency (time from beginning of the trial until the mouse falls off) to fall off the rotating rod. Six mice from each genotype, *Nefl*+*/*+;*pmn*, *Nefl*+/−;*pmn* and *Nefl*−*/*−;*pmn* were tested at a presymptomatic stage of postnatal day 21 and on three consecutive days when the mice showed signs of disease at the age of 27, 28 and 29 days both on a constant speed (8 rpm, 10 min maximum per test) and an accelerating speed rotarod (linear acceleration from 4 to 40 rpm within 2 min). The latency to fall off the rotating rod (in seconds) was recorded for each mouse for three trials spaced by 10 min each. The average latency for all the 9 trials for each mouse for three consecutive days (27–29) was plotted and used for further analysis. Forelimb grip strength of mice was tested using an automatic grip strength meter (Chatillon, Columbus Instruments) [47]. Mice were allowed to grasp a horizontal metal grid and pulled by their tail until the grip was released. The peak pull-force (in Newton) was recorded on the digital display. This test was performed for 6 trials per day at day 21 when disease was not apparent and for three consecutive days (age of 27, 28 and 29 days) when the mice showed signs of disease. Average grip strength from day 21 and 3 days at disease stages is plotted. The behavior tests were performed during the light cycle. All tests were performed on each mouse with a time gap of at least 1 h.

### Primary motoneuron culture

Lumbar spinal cord was dissected from embryonic day 13.5 mice as described [78]. Isolated spinal cord from each embryo was cleaned and trypsinized at 37 °C for 15 min in HBSS, containing trypsin at a final concentration of 0.1 %. Trypsinization reaction was stopped by adding trypsin inhibitor (Sigma-Aldrich; T9128) to a final concentration of 0.1 % and digested tissues were gently triturated to obtain single cells. Panning plates were prepared by coating 24-well Nunclon™ surface dishes with antibody against p75^NTR^, clone MLR2 (a gift from R. Rush, Flinders University, Adelaide, Australia; [61] and also commercially available through Biosensis (M-009-100) and Abcam (ab61425)), diluted to a final concentration of 5 ng/ml. Cells were transferred to panning dishes for 45 min where motoneurons expressing p75^NTR^ receptor attach to the antibody coated surface of the dish. Enriched motoneurons were then plated on polyornithine and laminin-111 (catalog no. 23017–015, lot no. 1347084; Invitrogen) coated glass coverslips (Marienfeld) or dishes. These cells were cultured at 37 °C temperature, 5 % CO_2_ in neurobasal medium (Invitrogen) containing 500 μM GlutaMAX (Invitrogen), 2 % horse serum (Linaris), 2 % B-27 supplement (Invitrogen), and the neurotrophic factor BDNF (5 ng/ml). Culture medium was changed on day 1, 3 and 5 after plating. 2000 cells were plated per 10 mm coverslip for measurements of axon length, 2000 cells per dish were plated for electron microscopic analyses and 5000 cells per 25 mm coverslip were plated for SIM.

### Electron microscopy of primary motoneurons

Motoneurons were cultured on special Falcon dishes (BD Falcon™-Dish 35 × 10 mm non-TC Petri EZGrip 500cas—BD Biosciences) for 7 days following the motoneuron culture protocol as described above. These motoneurons were fixed with 2.5 % glutaraldehyde and 0.8 % tannic acid in 0.1 M cacodylate buffer pH 7.5 (CB), for 5 min at 37 °C followed by 90 min at room temperature. Neurons were then washed three times with CB and treated with 1 % OsO_4_ in CB for 1 h. Cells were subjected to dehydration in 30, 50 % ethanol for 5 min each, followed by 30 min 0.5 % uranyl acetate in 70 % ethanol, followed by 90, 96, 100 % ethanol for 5 min each, and were finally embedded in a thin sheet of Epon (Serva, Heidelberg, Germany) resin. After polymerization, the resin sheets were stained with methylene blue for light microscopic identification of motoneurons. Resin pieces containing identified motoneurons were mounted on empty Epon blocks, ultrathin sections of ca. 80 nm were prepared, transferred to Formvar-coated nickel grids and contrasted with uranyl acetate and lead citrate [59]. Electron micrographs were obtained with a transmission electron microscope (LEO 912 AB; Carl Zeiss). Intermediate filaments have a diameter of 11.29 ± 0.46 nm. For measuring the IF density, 12 parallel lines at a distance of 173 nm, perpendicular to the long axis of the axon were drawn on the micrograph and the number of intermediate filaments intersecting each line was counted [12]; for proximal axons (within 50 μm distance to the cell body), distal axons (less than 100 μm distance to the axon tip), and intermediate parts (in-between these two regions). Width of the axon was measured for each line and IF density was calculated by dividing the mean number of IF intersection by mean axon width.

### Electron microscopy of phrenic nerves

Mice were killed by excessive CO_2_ exposure and transcardially perfused with a mixture of 4 % paraformaldehyde (PFA) and 2 % glutaraldehyde in CB. Distal phrenic nerves were collected and postfixed in the same fixative overnight at 4 °C. On the next day, nerves were washed with CB and treated with 2 % OsO4 in CB for 2 h, dehydrated in an ascending concentration of ethanol and embedded in Epon. Transverse ultrathin sections of the nerve were transferred to Formvar-coated nickel grids and contrasted with uranyl acetate and lead citrate. Electron micrographs were obtained with a transmission electron microscope (LEO 912 AB; Carl Zeiss). At least 3 mice from each genotype were used and microtubules were counted in transverse sections of axons from each mouse. Area of the axon was calculated using the ImageJ software (NIH).

### Immunocytochemistry and light microscopic analysis

Motoneurons cultured for 3 or 7 days were fixed with 4 % PFA (freshly prepared) for 20 min at room temperature. PFA was washed out with PBS by 3 washes for 5 min each. Fixed neurons were treated with blocking solution (0.3 % Triton X-100, 0.1 % Tween-20, 10 % horse serum in PBS) for 30 min. Primary antibodies diluted in blocking solution were added onto the neurons and incubated overnight at 4 °C. On the following day, neurons were washed three times with washing solution (0.1 % Triton X-100, 0.1 % Tween-20 in PBS), 10 min each wash and incubated for 1 h with the corresponding fluorescently labeled secondary antibodies at room temperature. Cells were washed with washing solution three times for 10 min each wash and mounted on object glass slides using aqua-polymount (Polysciences) and imaged under a confocal microscope (SP2; Leica) using a HC PL-APO 20×/0.70 objective. Axon length was measured using LAS AF software (Leica) by observers who were blind with respect to the genotype.

### Structured illumination microscopy

For structured illumination microscopy (SIM) analysis, motoneurons were cultured for 3 days and fixed with the above described protocols. Neurons were labeled by indirect immunofluorescence using secondary antibodies labeled with Alexa Fluor 488, Cy3 and Cy5. Specimens were imaged using a SIM Zeiss ELYRA S.1 microscope system with a 63×/1.40 oil immersion objective in x–y–z stacks. Raw images (16 bit) were processed to reconstruct high-resolution information using the provided commercial software package (Zeiss). Three-color images were aligned using a transformation matrix and were later processed with ImageJ. Shown are maximum-intensity projections of 5 z-stacks.

### Western blot analysis

Sciatic nerves from 34-day-old mice were isolated and lysed with a glass–glass homogenizer in RIPA buffer (50 mM Tris pH 7.4, 150 mM NaCl, 1 % Triton X-100, 0.1 % SDS, 2 mM EDTA, protease inhibitor, 0.5 % sodium deoxycholate, 1 mM NaF, 10 mM sodium pyrophosphate, 1 µM okadaic acid and 2 mM sodium orthovanadate). Concentration of protein was calculated using bicinchoninic acid assay (BCA assay) and lysate was boiled in Laemmli buffer for 10 min at 99 °C. Equal amount of protein from different samples were subjected to SDS-PAGE and transferred to nitrocellulose membranes. After incubation with the desired antibodies, membranes were developed using ECL or ECL advance (GE Healthcare). Obtained blots were scanned and band intensities were quantified by densitometry analysis with ImageJ (NIH).

### Immunoprecipitation

Sciatic nerves or NSC34 cells were lysed in IP buffer (50 mM Tris pH 7.4, 150 mM NaCl, 1 % Triton X-100, 2 mM EDTA, Protease inhibitor, 1 mM NaF, 10 mM sodium pyrophosphate, 1 µM okadaic acid, 2 mM sodium orthovanadate) using a glass–glass homogenizer (only for sciatic nerves lysis) and at least 200 µg of protein lysate was incubated with 5 µl anti-stathmin (rabbit) antibody, overnight at 4 °C on a rotating wheel. The protein-antibody mix was incubated with pre-equilibrated Protein A-agarose beads (Roche) for 1 h at 4 °C on a rotating wheel. Protein coupled beads were pelleted by centrifugation at 500 rpm for 3 min at 4 °C and supernatant was stored at −20 °C. Beads were washed three times with IP buffer and proteins were eluted by boiling the beads with 2X Laemmli buffer at 99 °C for 10 min. Eluted proteins were loaded on a SDS-PAGE and immunoblotting was done for stathmin, Stat3 and anti-NFL. The vector used for overexpression of NFL under a pRc/CMV promoter was supplied from Dr. JP Julien.

### Microtubule-regrowth assay

An established MT regrowth assay [1] with following modifications was used to study the MT regrowth in cultured motoneurons. 6000 cells were plated on polyornithine-laminin coated 12 mm coverslips and incubated at 37 °C for 1 h. Cells were then provided with full medium (2 % horse serum, 1X B-27 and 5 ng BDNF) containing 10 µM nocodazole to depolymerize the microtubule network. After 6 h of depolymerization, cells were washed 5 times with warm neurobasal (NB) medium and incubated at 37 °C for 5 min with 500 µl of warm NB to investigate the regrowth of microtubules. These cells were then washed with MT stabilizing buffer PHEM (60 mM Pipes, 25 mM Hepes, 10 mM EGTA, and 2 mM MgCl_2_) and MTs were extracted by adding PHEM with 0.5 % Triton X-100 and 10 µM paclitaxel for 3 min at 37 °C. Cells were rinsed with PHEM and fixed in 4 % PFA + PHEM (1:1). These motoneurons were then stained for α-tubulin to label polymerized MT and γ-tubulin to label the microtubule organizing center (MTOC). Images were taken at a SP5 confocal microscope (Leica, Bensheim, Germany) with a 63X oil objective, 4X magnification and 1.35 NA Image J was used to quantify the regrowth of microtubules. After background subtraction of radius 50, similar threshold was set for all the images and Sholl analysis was performed to quantify the number of microtubule intersections at a step size of 1 µm. Total length of MT for each cell was determined by multiplying the number of intersections at each step by its distance from the MTOC, starting from the periphery to the center. Mean length was calculated by summing up the length of MTs obtained and divided by the total number of MTs measured: Σ ((*nx* − *(nx* + 1)) × ∆MTOCx)/*N*, where *n* is the number of intersections, *x* is the circle of interest, *x* + 1 is the next outer circle, ∆MTOCx is the distance from MTOC to the circle of interest, and *N* is the total number of MTs.

### Reverse transcription, primer selection and qPCR

RNA from sciatic nerves of 1-month-old mice was isolated using the trizol RNA isolation method. cDNA was prepared and qPCRs performed with a Lightcycler 1.5 (Roche) with FastStart DNA master SYBR green1 reagents, using kinetic PCR cycles. Efficiency-controlled relative expression levels were calculated. Intron-spanning primers were selected with Oligo 6.0 software (MedProbe) and PCR conditions were optimized. Reactions were performed in glass capillaries in a volume of 20 µl. PCR products were analyzed by melting curve analysis. Primers and PCR targets: expression of housekeeping genes 5.8sRNA fw: 5′-GCGCTAGCTGCGAGAATTAATGTG-3′; rev-5′-CAAGTGCGTTCGAAGTGTCGATGA-3′ and mHPRT1 (NM_013556) fw: 5′-TTATGCCGAGGATTTGGAA-3′; rev-5′-ACAGAGGGCCACAATGTGAT-3′; 118 bp, intron-spanning, were used for the relative quantification. mNefl (NM_010910) fw: 5′-CTAAGACCCTGGAGATCGAAGCC-3′; rev-5′-GCTCTTCGTGCTTCTCAGCTCATT-3′; 149 bp, intron spanning. mStmn1 (NM_019641) fw: 5′-GCGCTTGCGAGAGAAGGACA-3′; rev-5′-CTCGGGACAACTTAGTCAGCCTCA-3′; 99 bp, intron spanning.

### Statistical analysis

Statistical analyses were performed using the GraphPad Prism 4.02 software (GraphPad, San Diego, CA, USA). A log rank test was used to test for the significance of differences in survival of mice as shown in the Kaplan–Meier curves in Fig. 2a. Parametric tests were used for normally distributed data and non-parametric tests for data which were not normally distributed. All tests were two-tailed unless otherwise mentioned. All data are expressed as mean ± SEM. Final processing of all images was performed with ImageJ (Rasband, WS, ImageJ, US National Institutes of Health, Bethesda, Maryland, USA, http://imagej.nih.gov/ij/, 1997–2015), [65] and Photoshop CS5 (Adobe). Brightness and contrast were enhanced for better visualization.

## Results

### Increased intermediate filament levels in *pmn* mutant motoneurons

**Fig. 1:**
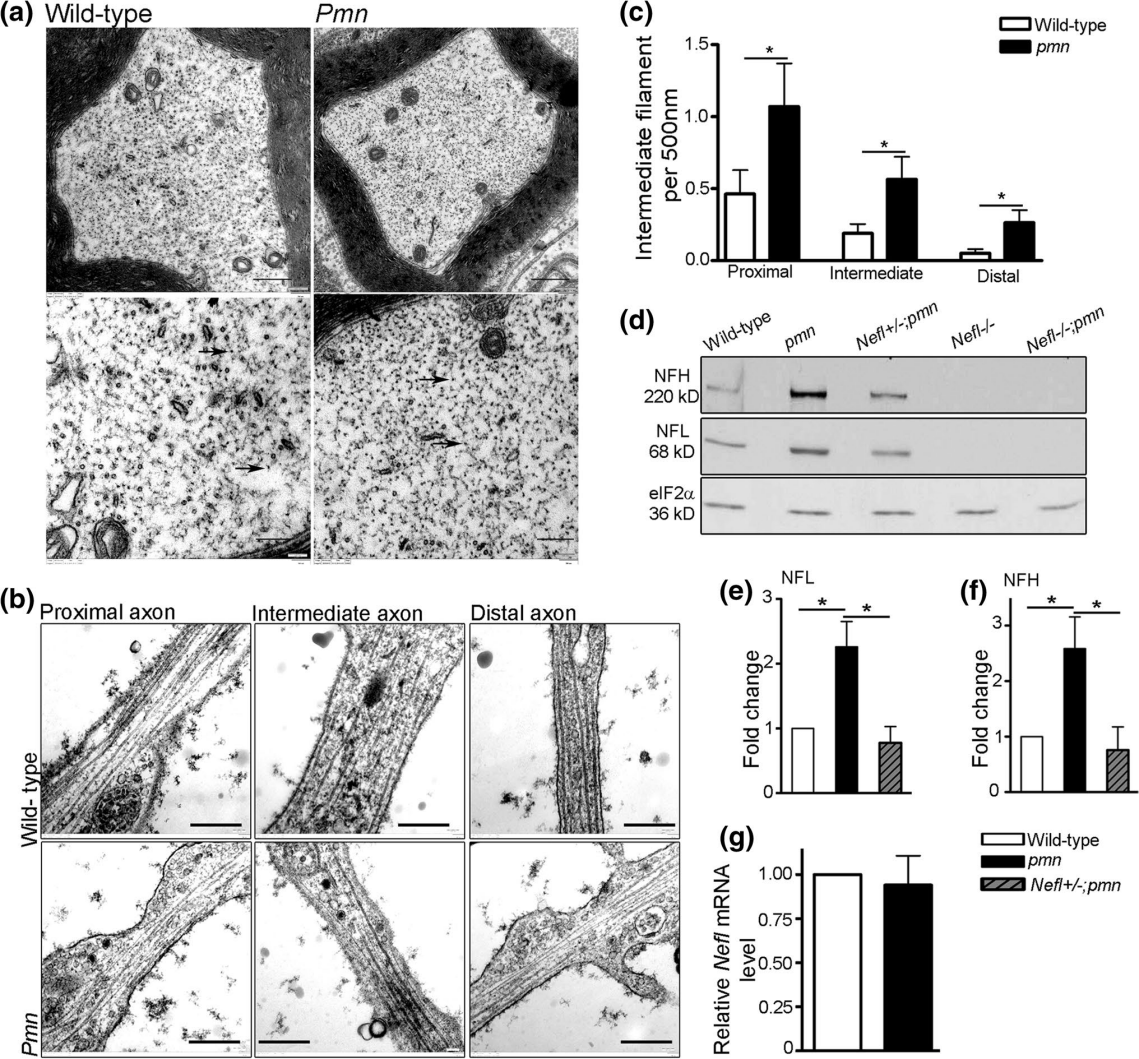
Analysis of intermediate filament (IF) levels in axons of *pmn* mutant mouse motoneurons. **a** Electron micrograph of cross sections of distal phrenic nerve from 34-day-old mice showed increased intermediate filaments in *pmn* axons compared to wild-type axons, *scale bar* 500 nm (*top*). *Bottom lane* shows the higher magnification of the respective image in the *top lane* (*scale bar* 200 nm). *Arrows* point to IF. **b** Electron micrograph showing axonal compartments of motoneurons cultured for 7 days. *Scale bar* 500 nm. **c** Quantification of number of IF in *pmn* axons showed an increase in proximal (*P* value = 0.0304), intermediate (*P* value = 0.0249) and distal (*P* value = 0.0336) compartments as compared to wild-type axons (Mann–Whitney one tailed test, *n* = 16 wild-type motoneurons and 19 *pmn* motoneurons from 3 independent experiments). **d** Western blot analysis of sciatic nerve lysate from 34-day-old mice. **e**, **f** *Pmn* mutant mouse nerves show increased levels of **e** NFL (*t* = 3.210, *P* = 0.0326) and **f** NFH (*t* = 2.781, *P* = 0.0498) proteins as compared to the wild type. *Bars* represent mean ± SEM, (*n* = 5 wild-type and *pmn* and *n* = 3 *Nefl*+/−;*pmn* mice analyzed, *P < 0.05; one sample *t* test). **g** Expression levels of *Nefl* mRNA in sciatic nerve extracts of 28- to 30-day-old *pmn* is not changed compared to wild type. Quantification was performed by normalizing with HPRT1 as housekeeping gene. *Bars* represent mean ± SEM (*n* = 3)

Axonal accumulation of neurofilaments is a pathological hallmark in many types of motoneuron disease. *Pmn* mutant mice suffer from a severe form of motoneuron disease which starts in third postnatal week and leads to death within 3–4 weeks [64]. In order to investigate the axonal pathology in *pmn* mice, we first analyzed the ultrastructure of phrenic nerves from 34-day-old *pmn* mice by electron microscopy. We observed a marked increase in number and density of intermediate filaments with typical diameter of approx. 10 nm in cross sections of phrenic nerve from *pmn* mutant mice (Fig. 1a). We then analyzed the ultrastructure of isolated embryonic motoneurons which were cultured for 7 days in vitro. Analysis of proximal, intermediate and distal compartments of *pmn* motor axons showed an increase in number of intermediate filaments in all the compartments as compared to the wild-type axons (Fig. 1b, c). Next, we analyzed the levels of neurofilament proteins in peripheral nerves of *pmn* mutant mice. For this purpose, sciatic nerves were isolated from 34-day-old *pmn* mutant mice, representing an advanced stage of disease, and subjected to Western blot analysis for NFL and NFH protein. *Pmn* mutant mice showed a significant increase in NFL and NFH protein levels in comparison to wild-type mice (Fig. 1d–f). Equal loading was controlled by determining eIF2α levels. The increase of NFL expression did not occur on the transcriptional level (Fig. 1g), indicating that altered translation or altered stability of neurofilaments accounts for this observation.

### NFL depletion prolongs life span and improves motor performance in *pmn* mutant mice

In order to study the role of increased NFL in *pmn* disease phenotype, mutant mice were crossbred with *Nefl* knockout mice. Deletion of one *Nefl* allele reduced the levels of NFL in *Nefl*+/−;*pmn* littermates to control levels, as observed in wild-type mice (Fig. 1d, e). Also, the increased levels of NFH protein in *pmn* mice were normalized to wild-type levels in *Nefl*+/−;*pmn* mice (Fig. 1d, f).

**Fig. 2:**
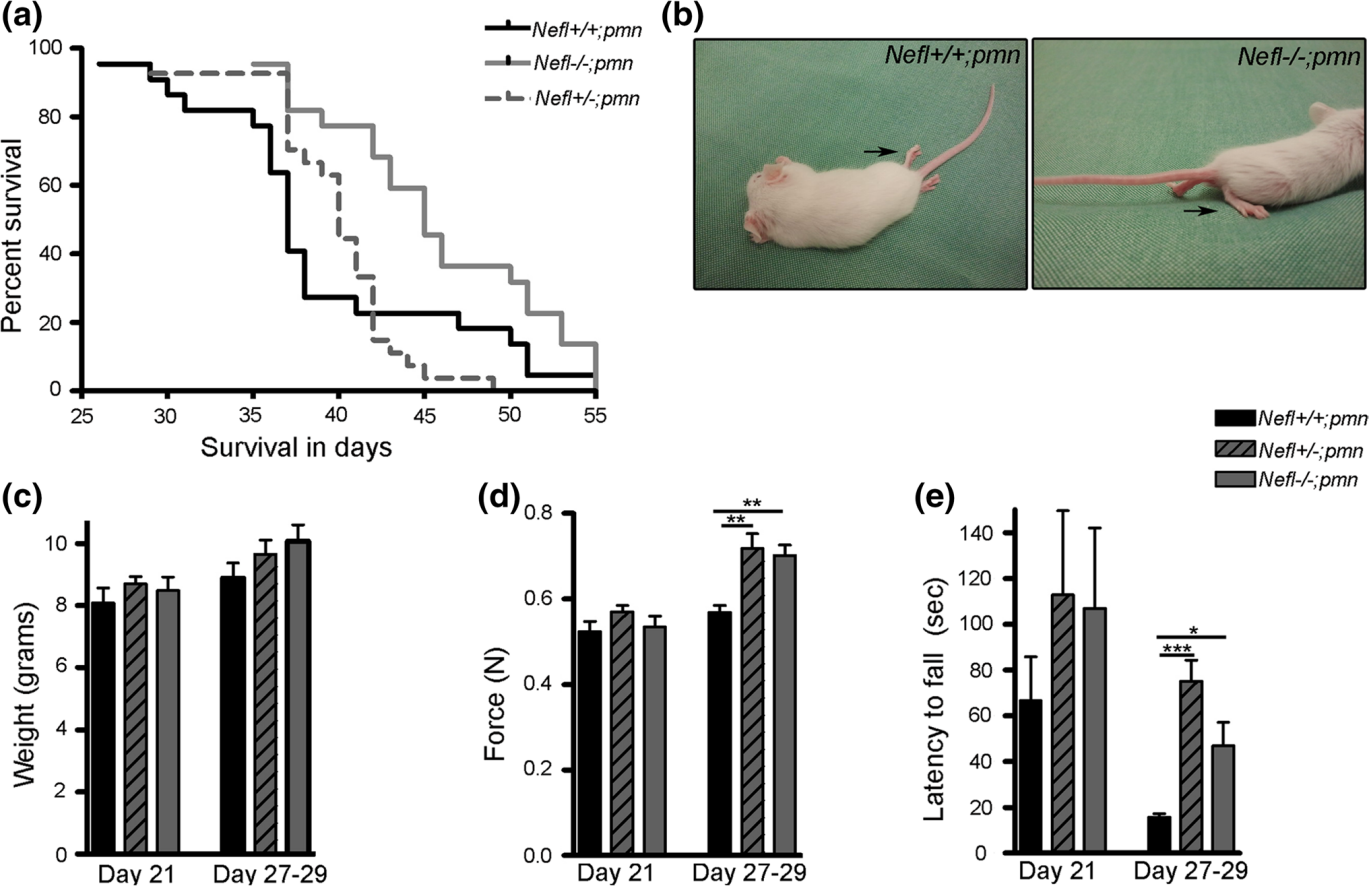
Life-span and phenotype of *pmn* mutant mice lacking NFL. **a** Kaplan–Meier curve for comparison of life span of mutant mice (log rank test; *n* = 22 *pmn* and *n* = 22 *Nefl*−*/*−;*pmn* mice and *n* = 27 *Nefl*+/−;*pmn* mice analyzed, *P* = 0.0110, *χ* ^*2*^ = 6.460). **b** Representative images of 29-day-old mice from the same litter. *Arrows* show hind limbs of these mice. **c** Mice were weighed at postnatal day 21 and 27–29 and showed no significant difference in average weight. **d** Peak forelimb grip strength in Newton (N) of mice measured at day 21 showed no significant difference, but at 27–29 *Nefl*+/−;*pmn* (*P* < 0.01; *t* = 4.007) and *Nefl*−*/*−;*pmn* (*P* < 0.01; *t* = 3.578) mice exhibited higher grip strength as compared to *Nefl*+*/*+;*pmn* mice. **e** On an accelerating speed rotarod, *Nefl*−*/*−;*pmn* (*P* < 0.05; *t* = 2.735) and *Nefl*+/−;*pmn* (P < 0.001; *t* = 5.206) mice showed an increase in the latency to fall as compared to *Nefl*+*/*+;*pmn* mice. **c**–**e** *Bars* represent mean ± SEM (one-way ANOVA and Bonferroni’s post hoc test, *n* = 6 mice per genotype, *P < 0.05, **P < 0.01, ***P < 0.001). *Bars* show average of the tests on postnatal day 21 and 27, 28 and 29 days

To investigate the effect of increased neurofilament expression on the neurodegenerative process in *pmn* mutant mice, we generated *pmn* mice completely lacking neurofilament light chain. Western blot analysis of sciatic nerve protein extracts from 34-day-old mice confirmed the absence of NFL (Fig. 1d) in *Nefl*−*/*−;*pmn* mice. *Nefl* deletion *per se* does not reduce survival of the mice, and *Nefl* knockout mice do not show an overt disease phenotype [83]. We followed the life span of *pmn* mice that lack NFL. *Nefl*−*/*−;*pmn* mice showed a significant extension of life span with an average increase of ~21 % in median survival as compared to *Nefl*+/+;*pmn* mice (log rank test; *P* = 0.0110, *χ*^*2*^ = 6.460; Fig. 2a). Median survival of *Nefl*−*/*−;*pmn* (*n* = 22) mice was 45 days and it was significantly higher than the median survival of 37 days in *Nefl*+*/*+;*pmn* (*n* = 22 mice). Median survival of *Nefl*+*/*−;*pmn* (*n* = 27 mice) was 40 days. Of the 22 mice analyzed at postnatal day 41, 18 *Nefl*−*/*−;*pmn* mice were still alive. At the same time point only 6 out of 22 *Nefl*+*/*+;*pmn* were alive. Figure 2b shows the hind limb of *pmn* mice from the same litter with (left) and without (right) NFL at postnatal day 29. Notably, the *pmn* mouse with NFL exhibited completely atrophied hind limbs, at the same age when *Nefl*−*/*−;*pmn* showed still preserved hind limb motor function. The average weight of *pmn*, *Nefl*+*/*−;*pmn* and *Nefl*−*/*−;*pmn* mice did not differ significantly at day 21 which represents a presymptomatic or early disease stage or at 27–29 days which marks a period of rapid disease progression (Fig. 2b, c).

In order to assess the motor performance and disease progression in *Nefl*−*/*−;*pmn* mice, we tested the grip strength of six mice per genotype and their performance on the rotarod on day 21 at disease onset and for three consecutive days (age of 27, 28 and 29 day) during a phase of fast disease progression (Fig. 2d, e). No significant difference was observed in the grip strength or rotarod performance between the three genotypes (Fig. 2d, e; supplementary Figure 3) at postnatal day 21. At day 27–29, we found that both *Nefl*−*/*−;*pmn* and *Nefl*+*/*−;*pmn* mice exhibited higher grip strength as compared to *Nefl*+*/*+;*pmn* mice (Fig. 2d), using a grip strength meter. There was a tendency towards higher latency to fall off the accelerated rotarod on day 21 in *Nefl*−*/*−;*pmn* mutant mice but this difference was not statistically significant (*n* = 6–7 for each group, *P* > 0.05). At day 27–29, *Nefl*−*/*−;*pmn* and *Nefl*+/−;*pmn* mice showed an increase in the latency to fall off the rotating rod, both on an accelerating (Fig. 2e) and a constant speed rotarod (Suppl. Figure 1) as compared to *Nefl*+*/*+;*pmn* mice. On the accelerating speed rotarod *Nefl*−*/*−;*pmn* could stay up to 50 s on the rod when the rotating speed was increased to 40 rpm, in contrast to *Nefl*+*/*+*pmn* mice which could not sustain the higher rotating speed at day 27–29. Interestingly, *Nefl*+*/*−;*pmn* mice in which the NFL levels are comparable to wild-type mice performed better in the rotarod test (Suppl. Figure 1) than *Nefl*−*/*−;*pmn* mice that completely lack NFL. Hence, deletion of *Nefl* from *pmn* mice does not influence disease onset, but prolongs survival of these mice, improves motor performance and partially ameliorates the disease phenotype.

### NFL depletion increases microtubule number in motor nerves of *pmn* mice and rescues defective axon elongation in vitro

**Fig. 3:**
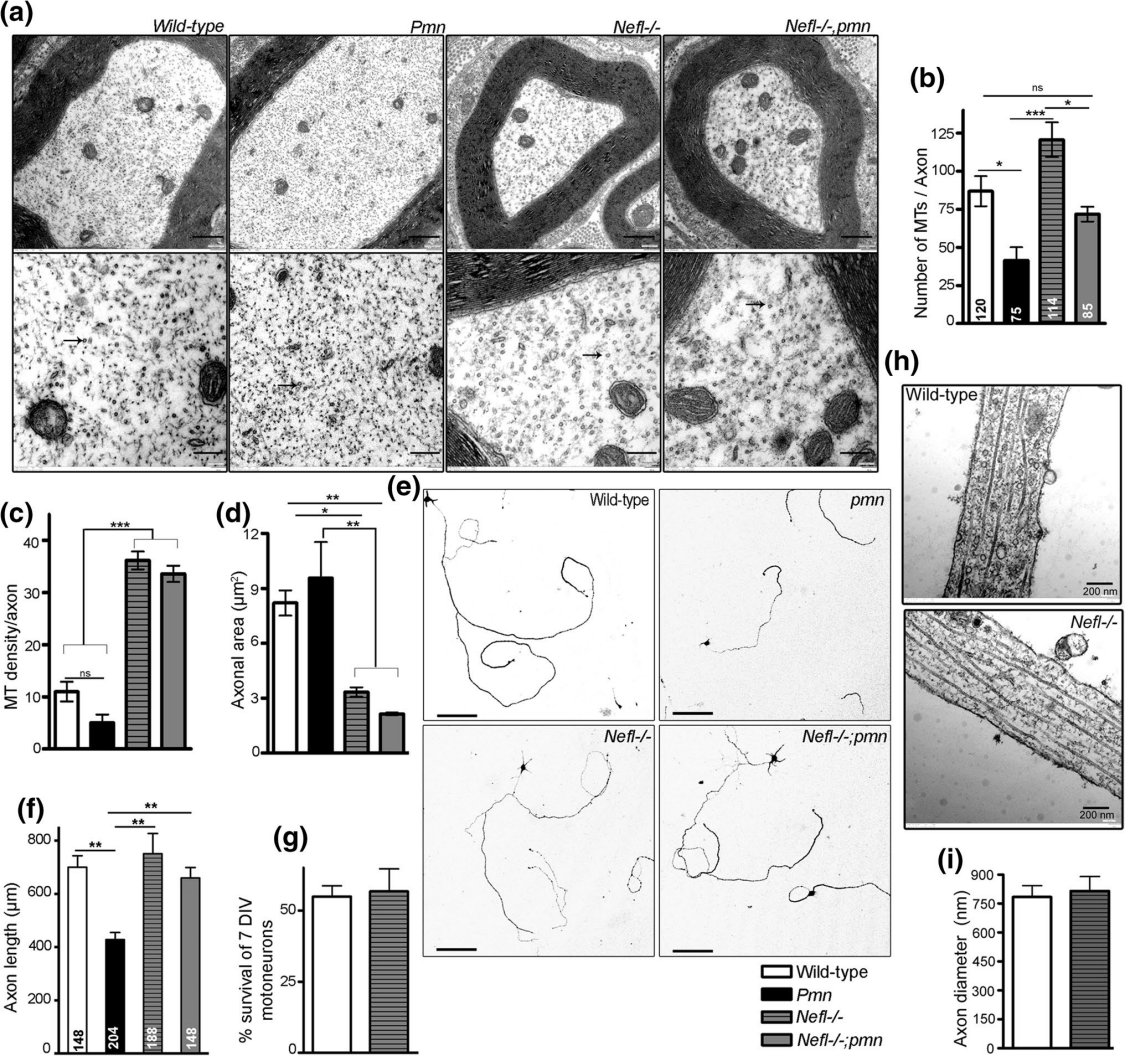
NFL depletion increases microtubule (MT) density and axon length in *pmn* mutant motoneurons. **a** Electron micrographs of distal phrenic nerve cross-sections from 34-day-old mice (*top*), *scale bar* 500 nm. *Bottom lane* shows the higher magnification of the respective image in the *top lane*, *scale bar* 200 nm. *Arrows* point to MTs. **b** Number of MTs per axon in *pmn* (*P* < 0.05; *t* = 3.640) decreased, whereas *Nefl*−*/*−;*pmn* mice (*P* > 0.05; *t* = 2.701) showed MT number comparable to wild-type mice. **c** Density of MTs per axon in *Nefl*−*/*− (*P* < 0.001; *t* = 10.05) and *Nefl*−*/*−;*pmn* (*P* < 0.001; *t* = 9.649) mice increased as compared to wild-type axons. **d** Cross-sectional area of *Nefl*−*/*− (*P* < 0.05; *t* = 3.416) and *Nefl*−*/*−;*pmn* (*P* < 0.01; *t* = 4.488) reduced as compared to wild-type and *pmn* axons (*n* = 3 *pmn* and *Nefl*−*/*− mice and *n* = 4 wild-type and *Nefl*−*/*−;*pmn* mice analyzed). **e** Representative images of motoneurons cultured for 7 days in vitro. Scale bar 100 µm. **f** *Pmn* motoneurons showed reduced axon length (*P* < 0.01; *t* = 6.845). *Nefl*−*/*−;*pmn* motoneurons grew significantly longer than *pmn* motoneurons (*P* < 0.01; *t* = 5.830) (*n* = 3 independent experiments). *Numbers* in the *bars* represents total number of motoneurons analyzed. *Bars* represent mean ± SEM (one-way ANOVA and Bonferroni’s post hoc test, *P < 0.05, **P < 0.01, ***P < 0.001). **g** *Bar graph* showing no change in percentage survival of motoneurons after 7 days in vitro culture. **h** Electron micrographs showing longitudinal section of axons of wild type and *Nefl*−*/*− motoneurons cultured for 7 days in vitro. **i** *Bar graph* showing quantitative analysis of axonal diameter of cultured embryonic motoneurons from wild-type (*n* = 7) and *Nefl*−*/*− (*n* = 9) mice, showing no change in width after 7 days in vitro. 2–3 sections of each proximal, intermediate and distal axon were analyzed for each motoneuron and the average of all sections from the three compartments is shown in the figure

*Pmn* mutant mice exhibit reduced axonal microtubule density in the distal part of phrenic nerves [46, 63]. Similarly, cultured motoneurons from *pmn* mutant mice show reduced axonal microtubule density in the proximal part of the axons [66]. On the other hand, *Nefl*−*/*− mice show increased microtubule numbers in axons within spinal cord and higher α-tubulin protein levels in the cerebral cortex [28]. In order to study the microtubule dynamics in *Nefl*−*/*−;*pmn* mice, we analyzed the ultrastructure of distal phrenic nerves in 34-day-old wild-type, *pmn, Nefl*−*/*− *and Nefl*−*/*−;*pmn* mice. Confirming previous analyses [46, 62] we observed a reduction in the number of microtubules per axon in *pmn* mutant mice (Fig. 3a, b) as compared to wild-type mice (wild-type 86.91 ± 9.793 and *pmn* 41.52 ± 8.585) (*P* < 0.05; *t* = 3.640; one-way ANOVA). Deletion of *Nefl* prevented the loss of microtubules per axon in phrenic nerves of *pmn* mutant mice, and *Nefl*−*/*−;*pmn* (71.83 ± 4.858) axons showed microtubule numbers comparable to wild-type (Fig. 3a, b; *P* > 0.05; *t* = 1.306; one-way ANOVA). However, the total number of microtubules per axon was not significantly higher in *Nefl*−*/*− mice (120.6 ± 11.47) compared to axons from control wild-type mice (86.91 ± 9.793; *P* > 0.05; *t* = 2.701), indicating that depletion of NFL resulted in maintenance or stabilization of microtubules but not in the generation of additional microtubules. *Pmn* pathology did not alter axonal diameter and therefore the cross-sectional area of axons in phrenic nerve of *pmn* mice remained unaffected (Fig. 3d) at postnatal day 34. In contrast, NFL appeared as a major modulator of axon caliber [24]. Confirming previous studies, we observed that NFL depletion led to a drastic reduction in the cross-sectional area of the axons in *Nefl*−*/*− and *Nefl*−*/*−;*pmn* axons in phrenic nerves of 34-day-old postnatal mice as compared to wild-type controls (Fig. 3d). Consequently, the density of microtubules per axon increased drastically in *Nefl*−*/*− and *Nefl*−*/*−;*pmn* nerves as compared to wild-type and *pmn* controls (Fig. 3c) in postnatal nerves at stages when these mice show clinical symptoms. These data suggest that NFL depletion prevents destabilization of microtubules in *pmn* mice and leads to an increased density of microtubules in axons by reducing the axonal caliber in both wild-type and *pmn* mutant mice.

Arrangement, distribution and organization of microtubules are essential for axonal outgrowth and elongation [14, 74]. Isolated motoneurons from *pmn* mutant mice show reduced axon outgrowth when cultured for 7 days in vitro [7], which could be rescued by CNTF application [66]. In order to study the effect of increased microtubule dynamics on axon elongation in *pmn* motoneurons lacking NFL, we measured and compared axon length of *Nefl*+*/*+;*pmn* and *Nefl*−*/*−;*pmn* mutant motoneurons cultured for 7 days in vitro in presence of BDNF. CNTF was not present in these cultures. *Pmn* motoneurons showed significantly shorter axons when cultured with BDNF (Fig. 3e, f), whereas axons from *Nefl*−*/*− motoneurons grew comparable to those of wild-type motoneurons. *Nefl* deletion completely rescued the defect in axon elongation of *pmn* mutant motoneurons, and *Nefl*−*/*−;*pmn* motoneurons grew significantly longer axons as compared to axons of *pmn* motoneurons, reaching axon lengths comparable to those of wild-type. Survival of *Nefl*−*/*− motoneurons after 7 days in vitro did not differ from that of wild-type motoneurons (Fig. 3g).

Previous studies [66] showed that the axon diameter of cultured *pmn* motoneurons does not differ from wild-type controls, which is in line with the observations made in this study with axons from phrenic nerves of the same mutant mice at postnatal day 34 (Fig. 3d). In order to test how axons were altered in *Nefl*−*/*− motoneurons, longitudinally sectioned motor axons were analyzed under the electron microscope. Proximal, intermediate and distal compartments of wild-type and *Nefl*−*/*− motor axons were used to measure the axonal diameter and the average diameter of wild-type and *Nefl*−*/*− axons is plotted. Axons of *Nefl*−*/*− motoneurons cultured for 7 days in vitro did not show changes in diameter as compared to wild type (Fig. 3h, i), indicating that the rescue effect observed in *Nefl*−*/*−;*pmn* mutant motoneurons is not a consequence of altered axon diameter.

### *Nefl* deletion rescues microtubule regrowth defect in cultured *pmn* motoneurons

**Fig. 4:**
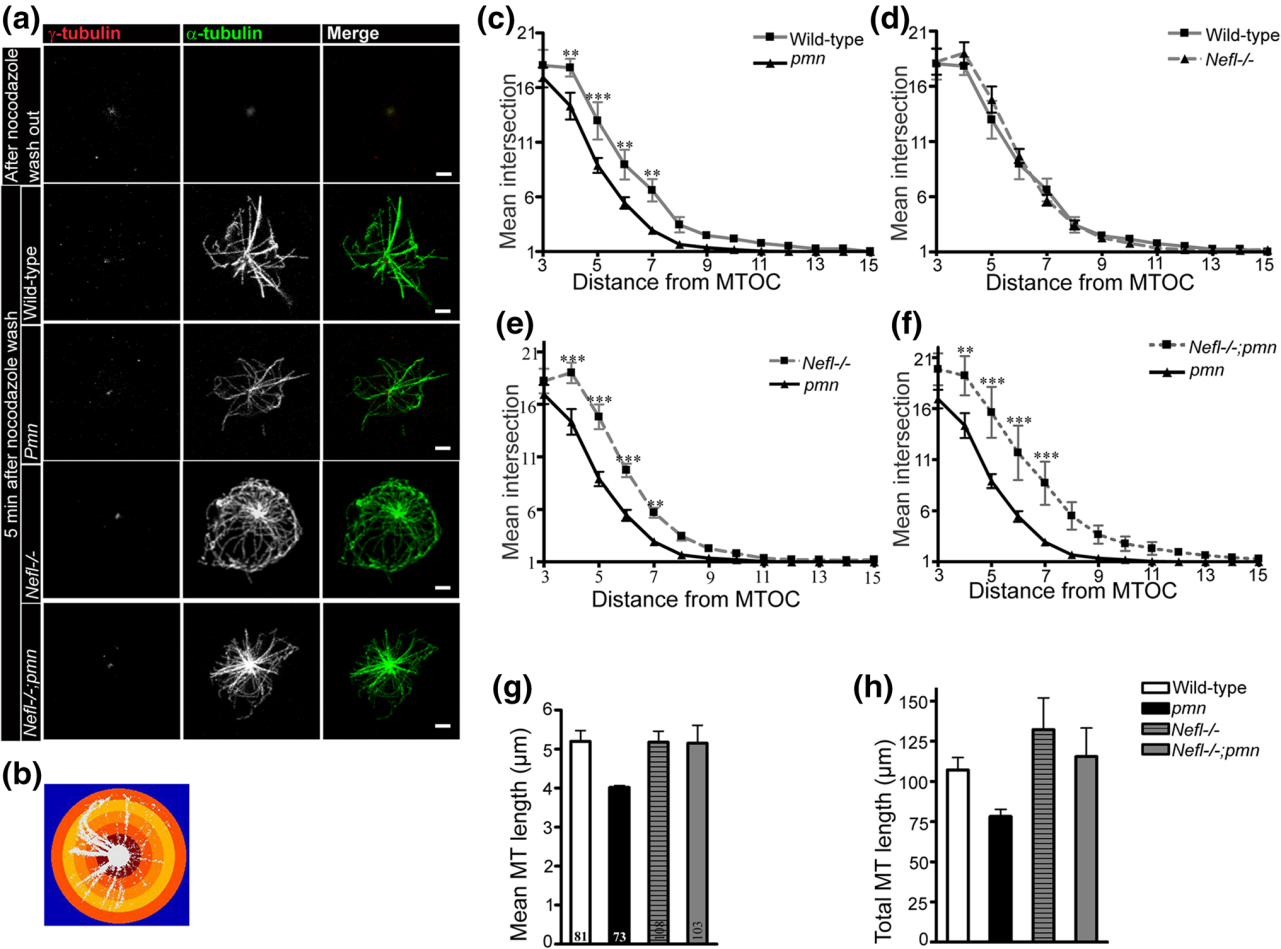
NFL depletion enhances microtubule (MT) regrowth in *pmn* mutant motoneurons in vitro. **a** Representative images of motoneurons showing depolymerized microtubules (MT) after nocodazole treatment and MT regrowth after 5 min of nocodazole washout. Microtubules (MT) are labeled using α-tubulin (*green*) and centrosome by γ-tubulin (*red*). *Bars* 2 µm. **b** Representative image of Sholl analysis performed to count the number of MTs with 1 µm concentric circles step. **c**–**f** Number of MT intersections (*y* axis) at increasing distance (*x* axis) from microtubule organizing center (MTOC) was counted. *Line graphs* showing mean ± SEM (two-way ANOVA with Bonferroni’s post hoc test, *n* = 4 independent experiments, **P < 0.01, ***P < 0.001). **g** Average length of MTs from each MTOC is plotted. **h** Total length of MTs regrown from each MTOC is plotted. *Bars* represent mean ± SEM

In order to study microtubule dynamics in *Nefl*−*/*− motoneurons, we investigated microtubule regrowth and polymerization in cultured motoneurons after complete disintegration of microtubules. For this purpose, an established protocol for MT regrowth after nocodazole treatment was used [1, 63, 66]. Isolated E13.5 motoneurons were plated in culture dishes for 1 h and the established microtubule network was completely depolymerized by applying 10 µM nocodazole for 6 h. Nocodazole was then washed off and microtubules were allowed to regrow. Sholl analysis (Fig. 4a, b) was performed to quantify MT polymerization, average MT length and total length of MT after regrowth. *Pmn* mutant motoneurons showed reduced MT regrowth when compared to wild-type motoneurons, as reported previously [66] (Fig. 4a, c). *Nefl*−*/*− motoneurons showed no change in MT repolymerization and the MT intersections appeared comparable to wild-type neurons (Fig. 4a, d). On the other hand, *Nefl* deletion significantly increased MT regrowth and repolymerization in *pmn* motoneurons, and *Nefl*−*/*−;*pmn* motoneurons showed significantly higher microtubule regrowth than *pmn* motoneurons (Fig. 4a, f) when cultured with BDNF.

Next, we measured the average length of microtubules. For this purpose, length of all the microtubules growing from the microtubule organizing center (MTOC) was measured and average length was calculated, as reported previously [66]. *Pmn* mutant motoneurons exhibited shorter average microtubule length compared to wild-type neurons (Fig. 4g). *Nefl*−*/*− motoneurons showed microtubule length comparable to wild type. Average microtubule length within *Nefl*−*/*−;*pmn* motoneurons was longer than the length of microtubules in *pmn* motoneurons and reached levels comparable to wild type and *Nefl*−*/*− controls (Fig. 4g). We also measured the total length of microtubules and found that *Nefl*−*/*−;*pmn* motoneurons show an increase in total microtubule length originating from MTOC as compared *pmn* motoneurons, that showed reduced total microtubule growth (Fig. 4h).

### NFL interacts with Stat3–stathmin complex and NFL depletion increases Stat3–stathmin interaction

**Fig. 5:**
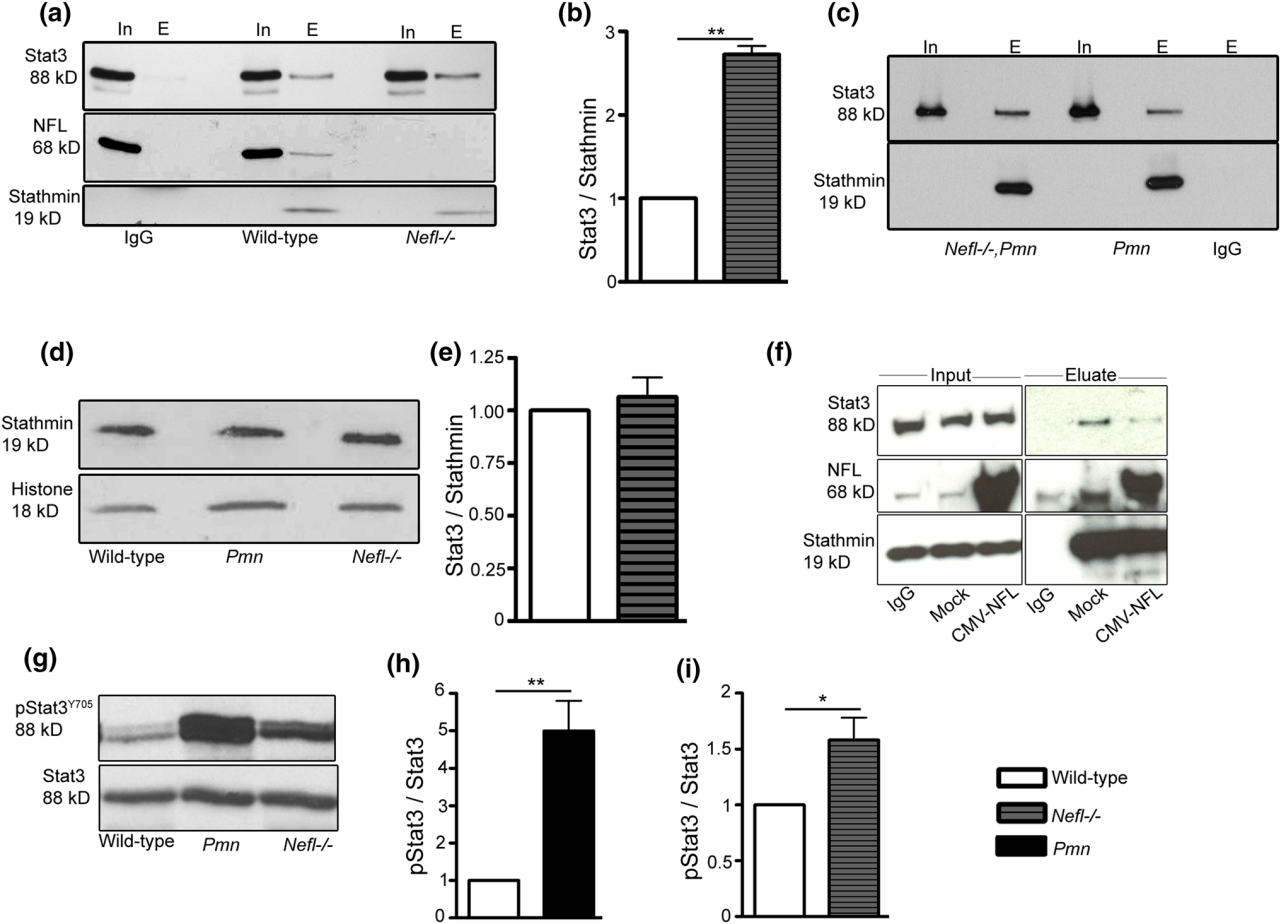
*Nefl* deletion increases Stat3–stathmin interaction. **a** Stathmin was immunoprecipitated from sciatic nerve extracts of 34-day-old mice. Western blot shows from *left* to right, input (Ip) and eluate (E) from wild-type and *Nefl*−*/*− mice nerve extracts after anti-IgG control and anti-stathmin pulldown. **b** Quantification of band intensities in the eluate showed an increased interaction of Stat3 and stathmin in *Nefl*−*/*− mice (*t* = 17.08; *P* = 0.0034). *Bars* represent mean ± SEM (one sample *t* test, *n* = 3 independent experiments). **c** *Nefl* deletion increases Stat3–stathmin interaction in sciatic nerves from *pmn* mutant mice. **d** Western blot analyses show similar levels of stathmin in the sciatic nerve extracts of 34-day-old wild-type, *pmn* and *Nefl*−*/*− mice. Histone levels were determined to ensure equal loading of proteins. **e** Quantification of Stat3 per stathmin levels in the sciatic nerve extracts (input before immunoprecipitation) from 34-day-old wild-type and *Nefl*−*/*− mice (*n* = 3 independent experiments). **f** Stathmin was immunoprecipitated from NSC-34 cell extracts. Western blot shows from left to right input for IgG, mock, and NFL overexpressing NSC-34 cells. *Right* blot shows eluate after IgG control pulldown and stathmin pulldown from NSC-34 cells after transfection with mock (only lipofectamine) or, NFL overexpression vector, respectively. **g** Western blot analysis reveals increased levels of phosphorylated Stat3 (at Y705) in the sciatic nerve extracts of 34-day-old *pmn* and *Nefl*−*/*− in comparison to wild-type mice. Quantification of pStat3 normalized by total Stat3 levels in **h** *pmn* mouse compared to wild type (*t* = 4.928; *P* = 0.0044; *n* = 6 independent experiments) and **i** in *Nefl*−*/*− mouse compared to the wild-type mouse (*t* = 2.865; *P* = 0.0242; *n* = 8 independent experiments). *Bars* represent mean ± SEM

CNTF-dependent restoration of axon length and maintenance of *pmn* motoneurons is mediated by local axonal activity of Stat3 [66]. Stat3 is activated upon CNTF application and the activated Stat3 interacts with stathmin thereby inhibiting its microtubule destabilizing activity and preventing the axonal degeneration in *pmn* motoneurons. Stat3–stathmin interaction has been shown to be increased in motoneurons upon CNTF application [66]. We therefore studied the interaction of Stat3–stathmin in *Nefl*−*/*− mice. We performed immunoprecipitation with protein lysates of sciatic nerves from 34-day-old wild-type and *Nefl*−*/*− mice via stathmin pulldown and investigated the interaction partners of stathmin. This pulldown experiment revealed that NFL also interacts with stathmin (Fig. 5a). Sciatic nerve lysates from *Nefl*−*/*− mice showed an increase in Stat3–stathmin interaction as compared to wild-type mice (Fig. 5a, b). Also in *pmn* mutant mice, NFL depletion led to an increase in Stat3–stathmin interaction in sciatic nerve extracts (Fig. 5c). In Western blots of sciatic nerve extracts, the levels of Stat3 (Fig. 5a) and stathmin (Fig. 5d) and also the ratio of Stat3/stathmin (Fig. 5e) were not altered, excluding the possibility that the enhanced Stat3–stathmin interaction is due to altered expression of any of these two proteins. Similarly, Stathmin2 levels were unchanged in *pmn* mutant sciatic nerves (Suppl. Figure 2a) and the mRNA levels for Stmn1 were not changed in the adult *pmn* or *Nefl*−*/*− mouse sciatic nerves (Supplementary Figure 2b, c). Hence, depletion of NFL increases the interaction of Stat3–stathmin that antagonizes the MT-destabilizing activity of stathmin [53].

In order to confirm the interaction of NFL and stathmin, we performed these immunoprecipitation experiments with NSC34 cells. For this purpose, cell lysates were subjected to stathmin pulldown and NFL and Stat3 interaction were studied by co-immunoprecipitation. Our data confirmed the interaction of NFL with stathmin in this cell culture system (Fig. 5f). Overexpression of NFL led to an increased interaction of NFL with stathmin and in parallel a decreased interaction with Stat3 (Fig. 5f).

Since the phosphorylation of Stat3 at Y705 plays an important role in its interaction with stathmin upon CNTF treatment [66], we studied the levels of phosphorylation of Stat3 in sciatic nerve extracts of 34-day-old *Nefl*−*/*− and *pmn* mice. The levels of phosphorylation showed an about fivefold increase in *pmn* nerve extracts (Fig. 5g, h). *Nefl*−*/*− nerve extracts also showed an increase in the phosphorylation at Y705, indicating that the increase in the stathmin/Stat3 interaction is mediated via activation of Stat3 (Fig. 5g , i).

### *Nefl* deletion affects the distribution and interaction of Stat3 and stathmin with tubulin in motoneurons

**Fig. 6:**
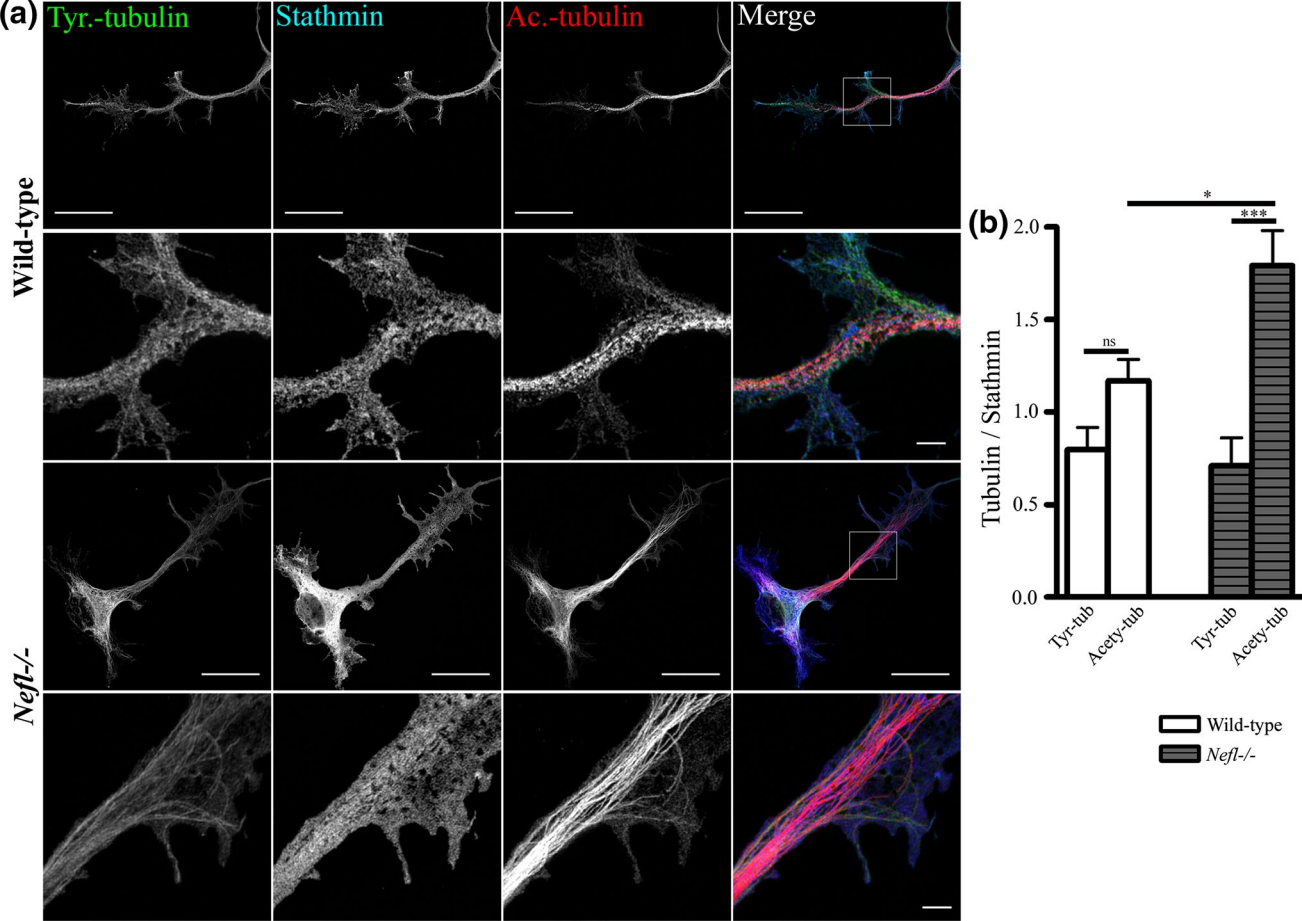
Levels of tyrosinated and acetylated tubulin in NFL-depleted motoneurons **a** Representative images of motoneurons showing increased acetylated microtubules in *Nefl*−*/*− motoneurons after 3 days in vitro culture. Neurons were stained with antibodies against tyrosinated α-tubulin (*green*), stathmin (*blue*) and acetylated α-tubulin (*red*). *Scale bar* 20 µm (*first and third lane*). *White square boxes* indicate the regions enlarged in the *second* and *fourth lane*, *scale bar* 2 µm. **b** Quantification of fluorescence intensities showed increased acetylated microtubules in *Nefl*−*/*− (*t* = 3.011) as compared to wild-type motoneurons, whereas tyrosinated microtubules remained unchanged. Intensity of stathmin is used as control. *Bars* represent mean ± SEM (one-way ANOVA, *n* = 10 motoneurons from three independent experiments, *P < 0.05, ***P < 0.001)

Dimers of α- and β-tubulin are the basic components of microtubules. These heterodimers undergo a variety of post-translational modifications which assign specific functions to the tubulin subunits. Acetylation of α-tubulin occurs in polymerized microtubules, and the acetylated tubulin is enriched in stable microtubules with a low turnover. On the other side, tyrosinated α-tubulin marks recently assembled more dynamic microtubules with a high turnover [77]. In neurons, acetylated microtubules are found in more proximal parts of axons where microtubules are thought to be stable, and they appear to be excluded from axon terminals [66, 80]. In the highly dynamic axonal growth cones and dendrites, tyrosinated tubulins are found [66]. *Pmn* motoneurons exhibit more highly dynamic MTs in axons and show higher levels of tyrosinated tubulins in axons (Suppl. Figure 3) as compared to wild-type motoneurons, whereas the levels of acetylated MTs are unchanged (Suppl. Figure 3 and [66]). We observed that NFL depletion increased the intensity of staining against acetylated tubulin in axons of motoneurons (Fig. 6a, b). In the same axons, levels of tyrosinated microtubules did not change. In summary, these data indicate that NFL depletion enhances the stability of microtubules, as could be seen by the higher levels of acetylation.

**Fig. 7:**
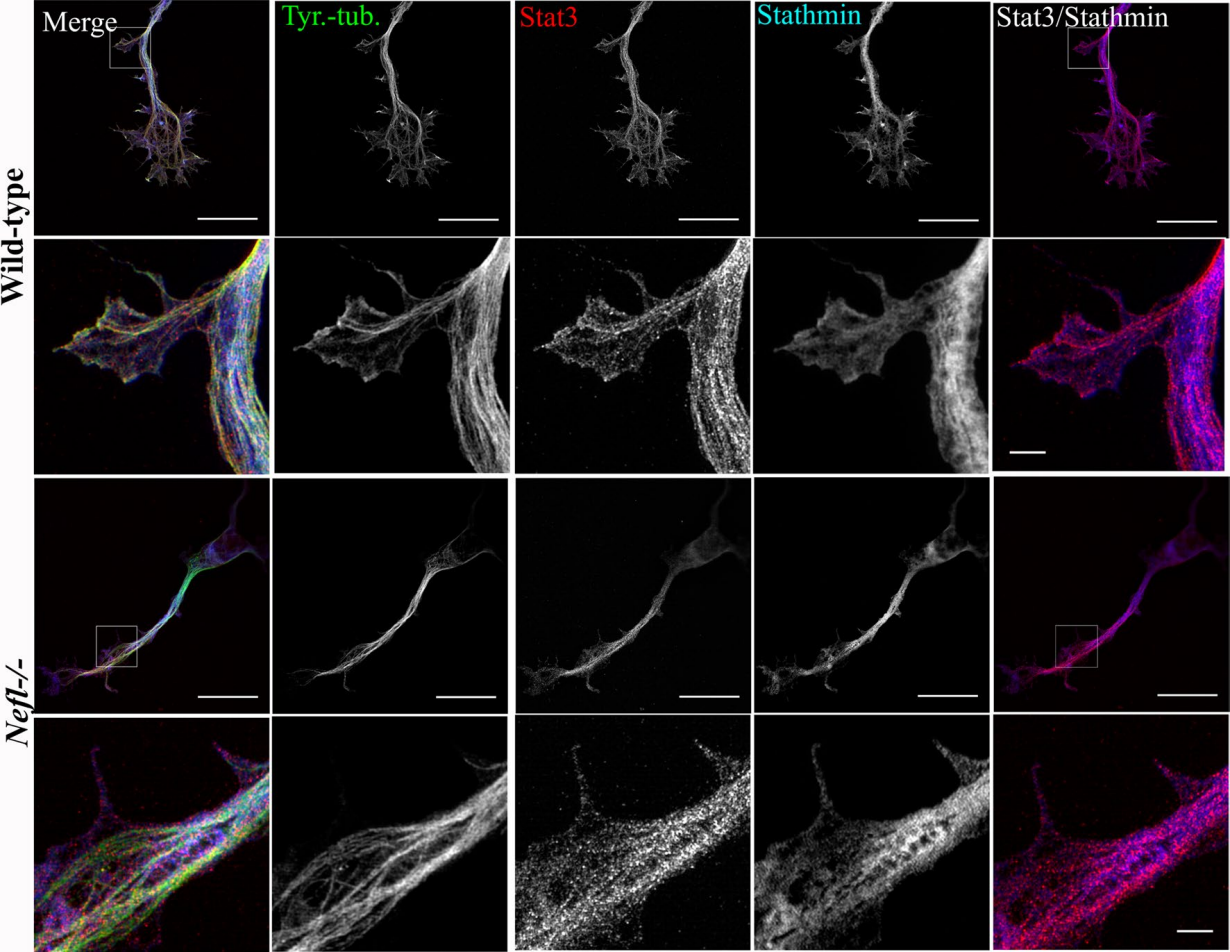
Distribution of Stat3 and stathmin in axons of wild-type and *Nefl*−*/*− motoneurons as revealed by high-resolution SIM. Motoneurons were cultured for 3 days in vitro. Representative images of wild-type and *Nefl*−*/*− motoneurons, stained with antibodies against tyrosinated α-tubulin (*green*), Stat3 (*red*), and stathmin (*blue*). The antibody against tyrosinated α-tubulin stains both soluble αβ-tubulin heterodimers and polymerized highly dynamic microtubules. Note that the colocalization of stathmin with Stat3 increases in *Nefl*−*/*− motoneurons (*right panel*). *Scale bar* 20 µm (*first and third lane*). *White square boxes* indicate the regions enlarged in the second and *fourth lane*, *scale bar* 2 µm

This effect could be explained by reduced activity of stathmin. Stathmin appears redistributed in axons of *pmn* mutant motoneurons, with higher levels of this protein in association with NFL (Suppl. Figure 4). Stathmin has two functions on microtubules dynamics. On the one side, it binds to heterodimers of α- and β-tubulin and prevents the polymerization of new microtubules. On the other side, it interacts with polymerized microtubules and induces their disassembly in microtubule catastrophe reactions [8]. In order to study this possibility, we determined the subcellular distribution of Stat3 and stathmin. In wild-type axons, Stat3 was observed to be co-localized with the tyrosinated tubulin whereas in the *Nefl*−*/*− axons Stat3 co-localized with stathmin (Fig. 7). These findings emphasize that the association of axonal Stat3 with stathmin modulates microtubule dynamics in the neurons. In addition, they support the data obtained by stathmin immunoprecipitation and Western blot analysis showing that NFL modulates the interaction of Stat3 and stathmin. Thus, NFL depletion increases the interaction between Stat3 and stathmin and stabilizes microtubules by reducing the MT destabilizing activity of stathmin.

## Discussion

In this study, we observed that NFL protein levels are at least twofold increased in *pmn* mutant motoneurons and that NFL depletion rescues defective axon growth in cultured motoneurons and prolongs survival of *pmn* mutant mice. This effect was found both in *Nefl*+/−;*pmn* motoneurons in which elevated NF expression was brought back to wild-type control levels and in *Nefl*−*/*−;*pmn* motoneurons in which axonal neurofilament was completely lost. In cultured *pmn* mutant motoneurons, NFL depletion also resulted in increased axon elongation and increased levels of acetylated tubulin, a marker for stable microtubules. Thus, NFL depletion modulates microtubule dynamics in a similar manner as observed after Stat3 activation. This effect was apparently due to increased phosphorylation of Stat3 and enhanced interaction of Stat3 with stathmin when NFL levels were reduced, resulting in enhanced stability of microtubules.

Accumulation of neurofilaments has been observed in a variety of neurodegenerative diseases and in corresponding mouse models [36, 42, 44]. Thus neurofilament pathology represents a point where pathomechanisms of distinct neurodegenerative disorders converge [40]. However, the reasons why NFL is upregulated in degenerating neurons and the precise mechanism how NFL accumulation participates in these neurodegenerative mechanisms are not fully understood so far. Several lines of evidence indicate that defective axonal transport leads to enhanced phosphorylation and accumulation of neurofilaments in axons [55]. The upregulation could also be a consequence of altered post-transcriptional control [73], or a consequence of defective intermediate filament degradation. The possibility of increased protein synthesis has been ruled out in degenerating neurons of ALS and Alzheimer disease patients in which *NEFL* mRNA levels are downregulated [3, 48, 71, 72, 81], whereas the transcript levels for *NEFM* or *NEFH* usually remain unchanged [81]. This indicates that both altered axonal transport, posttranscriptional mechanisms including deregulated translation or posttranslational mechanisms such as reduced degradation of NFL [60] could be responsible for the phenotype. Gigaxonin plays an essential role in the degradation of IFs [54] and the accumulated pool of IF in giant axonal neuropathy is lost upon Gigaxonin restoration in patient iPSC derived motoneurons [31], indicating that defective degradation could play a central role in this and other forms of neurodegenerative disorders.

Mouse models of motoneuron disease and other neurodegenerative disorders have provided support for the hypothesis that accumulation of NFL could be an early event in neurodegeneration. Overexpression of NFL, NFM or NFH transgenes causes NF aggregation and motoneuron dysfunction resembling motoneuron disease in mouse models [11, 21, 34, 82]. In mutant SOD1 mice, *Nefl* deletion delayed the onset of disease and slowed the disease progression [79], and in tau transgenic mouse it reduces the abnormal tau accumulation and motoneurons degeneration [28]. In *pmn* mutant mice, deletion of only one *Nefl* allele normalized NFL and NFH protein levels in sciatic nerves. Under these conditions, the motor function in *pmn* mutant mice was improved, thus providing evidence that the elevation of endogenous neurofilament levels contributes to axon destabilization and loss of motor function. Despite the drastic reduction of IFs and the resultant reduction in axon diameter in peripheral nerves, *Nefl*−*/*−;*pmn* mutant mice survived longer than *Nefl*+/−;*pmn* or *pmn* mutant mice. Thus, loss of IFs delays axon destabilization in *pmn* mutant mice.

Microtubules in *pmn* mutant mice are unstable, because the underlying gene defect leads to a massive reduction of αβ-tubulin heterodimers, the basic components of microtubules [46]. In cultured motoneurons, this leads to defective axon elongation [63, 66], and levels of tyrosinated microtubules are increased in the axons of these motoneurons. Intermediate filaments are anatomically and functionally linked with microtubules. Neurofilament interacts with tubulin [50] and stimulates microtubule polymerization in mature neurons [4]. Any disturbance of NF protein levels does not only affect assembly of NF fibers, it also influences microtubules [30]. NFM and NFH make cross bridges between adjacent NFs and microtubules [51]. Thus, also removal of NFM and NFH sidearms delays the disease in SOD1 mutant mice [43] in a similar manner as deletion of the *Nefl* gene in this mouse model [79]. Disease onset is also delayed in the same mouse model after treatment with microtubule stabilizing agents [19]. However, it has not been shown so far whether stabilization of microtubules contributes to the beneficial effects of NFL depletion or removal of NFM and NFH sidearms in SOD1 mice. The observation made in our study that levels of acetylated tubulin increase in axons of NFL-deficient motoneurons points to this possibility, and indicates that the beneficial effects of normalizing IF levels or those of massive reduction of functional IFs could be due to the stabilization of microtubules.

The levels of tyrosinated tubulin are increased in all compartments of the *pmn* mutant axons (Suppl. Figure 3) as observed in cultured motoneurons using light microscopy. The ultrastructural analyses of these *pmn* mutant axons (Fig. 1c) indicate that the increase of IF also occurs in all compartments with a gradient from proximal to distal, which however is similar in motoneurons from wild-type and *pmn* mutant mice. Thus the structural alterations in NFL upregulation do not go in parallel with dying back mechanisms in the cultured motoneurons, but they are thought to be of relevance for axonal transport processes in vivo which could contribute the degeneration of distal parts of the axons.

Stathmin plays a central role in the regulation of microtubule stability [8]. It acts in two distinct ways on microtubule dynamics. First, it destabilizes existing microtubules by inducing microtubule catastrophes in a dose-dependent fashion in vitro [2, 26, 45]. Second, it binds αβ-tubulin heterodimers and sequesters them in a way that microtubule polymerization is inhibited [32]. In vitro, a change in the pH of the buffer can lead to a shift in the role of stathmin from sequestration of tubulin affecting microtubule elongation to increase in microtubule catastrophes [27]. The N-terminal of stathmin is required for the catastrophic role, whereas the C-terminal is essential for the inhibition of MT-polymerization rate in vitro [25, 39]. These effects on regulating microtubule dynamics apparently are involved in plasticity processes when neurons change their shape and new neuronal connections are made, for example during learning and memory processes. Mice which lack stathmin-1 show severe defects in fear memory formation in the amygdala [69, 76] and this process correlates with high levels of stahmin-1 expression found in this brain region. Not much is known about how stathmin function is regulated, but its spatial distribution within cells seems to play a role [67]. Several members of the stathmin family are associated with membranous structures by palmitoylation that orients these proteins to specific subcellular compartment and thus restricts their subcellular distribution [8]. On the other hand, stathmin-1 lacks a palmitoylation signal, but this protein is not evenly distributed in the cytoplasm. In our study, we find stathmin-1 within axons, mostly colocalized with microtubules. High-resolution light microscopy with structured illumination microscopy (SIM) allowing resolution of structures down to nearly 100 nm reveals close proximity of stathmin with tyrosinated microtubules in the axons of wild-type motoneurons. This proximity seems to be increased in *Nefl*−*/*− motoneurons, and stathmin colocalization with Stat3 and microtubules also increases, as shown in Fig. 7, right panel. This increased interaction of stathmin with Stat3 is confirmed by biochemical immunoprecipitation assays. Neurofilament appears as part of this complex in pulldown assays, indicating that IFs play a role in the formation of complexes between Stat3 and stathmin. This could explain why IF depletion modulates microtubule stability.

In wild-type motoneurons, NFL depletion had a much more pronounced effect on stability of existing microtubules that are acetylated at relatively high levels when compared to microtubule regrowth after nocodazole treatment. In *pmn* mutant motoneurons in which availability of αβ-tubulin heterodimers is reduced, NFL depletion also restored defective microtubule regrowth after nocodazole treatment. This differential effect of NFL depletion could be explained by the reduced availability of αβ-tubulin heterodimers in *pmn* mutant motoneurons, which increases upon release of αβ-tubulin heterodimers when stathmin is inactivated by enhanced interaction with Stat3 [66]. Thus, the increased interaction of Stat3 and stathmin in NFL-depleted motoneurons enhances the capacity for microtubule regrowth and microtubule plasticity in motoneurons from *pmn* mutant mice. This also indicates that enhanced NFL levels in neurodegenerative diseases reduce the capacity for microtubule regrowth and microtubule plasticity. Our data provide evidence that the destabilizing activity of stathmin is enhanced when NFs are increased in *pmn* pathology and probably in other neurodegenerative disorders, and this could make a major contribution to axonal degeneration.

When this idea is followed up towards therapeutic implications, this would mean that catastrophe-inducing endogenous MT deregulators such as stathmin proteins should be functionally blocked in order to stabilize microtubules and to enhance stability of axons in conditions involving IF accumulation or microtubule destabilization. In neurons, altered MT-based transport and aggregation of proteins is generally associated with neurodegenerative disorders [57, 70]. Despite the fact that axons exhibit smaller diameter, the improvement in MT network in *Nefl*−*/*−;*pmn* motoneurons apparently stabilizes the axon and possibly also improves the transport of cargoes, leading to prolonged survival and delay in the decline of motor function.

In summary, our findings suggest that NF accumulation contributes to axonal destabilization in the *pmn* mouse model of motoneuron disease and possibly also other forms of neurodegenerative disorders. NFL depletion stabilizes the MT structure and leads to enhanced axon growth in *pmn* mutant motoneurons via increased activation of Stat3 by phosphorylation at Y705 and thereby increased Stat3–stathmin interaction. Thus, targeting the NFL accumulation in neurodegenerative diseases could be a target for therapy in neurodegenerative disorders.

## Electronic supplementary material

Below is the link to the electronic supplementary material.

**Fig. S1** On a constant speed rotarod, latency to drop is similar at day 21 in *pmn, Nefl*+/−*pmn* and in *Nefl*−*/*−;*pmn* mice but at days 27-29 *Nefl*+/−;*pmn* (*P* < 0.001; *t* = 4.663) and *Nefl*−*/*−;*pmn* (*P* > 0.05; *t* = 1.476) showed an increase in the latency to fall as compared to *Nefl*+*/*+;*pmn* mice. Bars represent mean ± SEM (one-way ANOVA and Bonferroni’s post hoc test, *n* = 6 mice per genotype, *P < 0.05, **P < 0.01, ***P < 0.001). Bars show average of the tests on postnatal day 21 in the left panel and 27, 28 and 29 days in the right panel. (TIFF 100 kb)

**Fig. S2** **a** Representative western blot using sciatic nerve lysate from 28 days old wild-type and *pmn* mouse showing no change in SCG10/stathmin2 levels in *pmn* mutant nerves compared to wild-type. Calnexin levels served as a control for equal loading. **b** Expression levels of *Stmn1* mRNA (from *stathmin 1* gene) in the sciatic nerve extracts of age matched adult *Nefl*−*/*− mice as compared to wild-type controls. **c** The expression level of *Stmn1* mRNA in age matched (28-30 days old) *pmn* and wild-type mice is represented by the bar graph. Quantification was performed by normalizing with HPRT1 or 5.8 s rRNA expression levels as housekeeping genes. Bars represent mean ± SEM (*n* = 3). (TIFF 52 kb)

**Fig. S3** Distribution of acetylated and tyrosinated tubulin in axons of wild-type and *pmn* motoneurons as revealed by high resolution SIM. Motoneurons were cultured for 3 days in vitro. Representative images of proximal (left) and distal axon (right) of wild-type and *pmn* motoneurons, stained with antibodies against tyrosinated α-tubulin (green), acetylated α-tubulin (red). Scale bar 50 µm. (TIFF 254 kb)

**Fig. S4** Subcellular localization of NFL and stathmin in wild-type (a) and *pmn* (b) motoneurons by high resolution SIM (upper panels, scale bar: 10 µm). Motoneurons were cultured for 3 days in vitro and stained with antibodies against NFL (red, DA2 clone, EnCor Biotechnology) and stathmin (green, rabbit monoclonal, Abcam). White square boxes indicate proximal (‘‘) and distal (‘‘‘) axonal sections which are enlarged in the corresponding lower panels (scale bar: 2 µm). (c) The degree of colocalization between NFL and stathmin appeared increased (MOC: *P* = 0.0231, *t* = 2.443, *df* = 22; PCC: *P* = 0.0294, *t* = 2.330, *df* = 22; unpaired *t* test) in *pmn* motor axons (*N* = 11, MOC = 0.60 ± 0.04, PCC = 0.51 ± 0.05) in comparison to wild-type motor axons (*N* = 13, MOC = 0.48 ± 0.03, PCC = 0.36 ± 0.04). Quantitative colocalization analysis was carried out in motor axons in a representative culture using the Manders Overlap Coefficient (MOC) and Pearson’s correlation coefficient (PCC) plugins of ImageJ. N is the number of motoneurons analyzed. (TIFF 2621 kb)
