# Supplementary figures and images for: Neurofilament depletion improves microtubule dynamics via modulation of Stat3/stathmin signaling
Source: Acta Neuropathol. 2016 Mar 28;132:93–110. doi: 10.1007/s00401-016-1564-y (PMC4911381; doi:10.1007/s00401-016-1564-y)

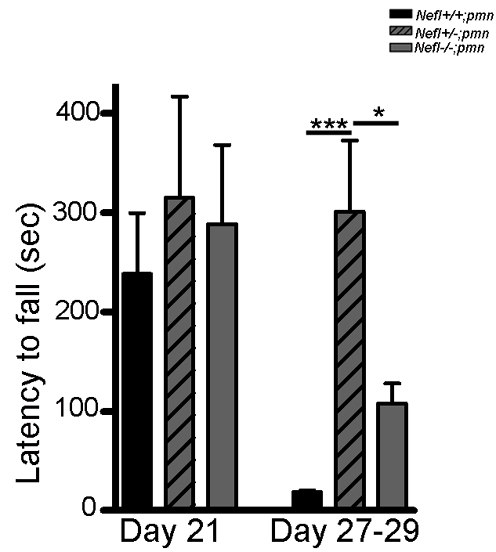

Supplement: Supplementary file 1 — Fig. S1 On a constant speed rotarod, latency to drop is similar at day 21 in pmn, Nefl+/−pmn and in Nefl−/−;pmn mice but at days 27-29 Nefl+/−;pmn (P < 0.001; t = 4.663) and Nefl−/−;pmn (P > 0.05; t = 1.476) showed an increase in the latency to fall as compared to Nefl+/+;pmn mice. Bars represent mean ± SEM (one-way ANOVA and Bonferroni’s post hoc test, n = 6 mice per genotype, *P < 0.05, **P < 0.01, ***P < 0.001). Bars show average of the tests on postnatal day 21 in the left panel and 27, 28 and 29 days in the right panel. (TIFF 100 kb) [file 401_2016_1564_MOESM1_ESM.tif]

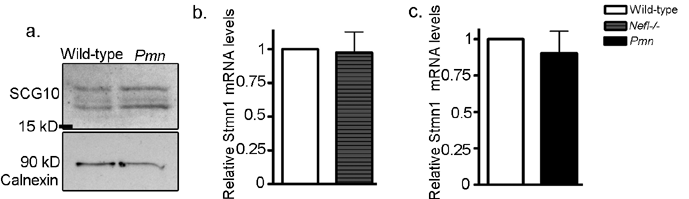

Supplement: Supplementary file 2 — Fig. S2 a Representative western blot using sciatic nerve lysate from 28 days old wild-type and pmn mouse showing no change in SCG10/stathmin2 levels in pmn mutant nerves compared to wild-type. Calnexin levels served as a control for equal loading. b Expression levels of Stmn1 mRNA (from stathmin 1 gene) in the sciatic nerve extracts of age matched adult Nefl−/− mice as compared to wild-type controls. c The expression level of Stmn1 mRNA in age matched (28-30 days old) pmn and wild-type mice is represented by the bar graph. Quantification was performed by normalizing with HPRT1 or 5.8 s rRNA expression levels as housekeeping genes. Bars represent mean ± SEM (n = 3). (TIFF 52 kb) [file 401_2016_1564_MOESM2_ESM.tif]

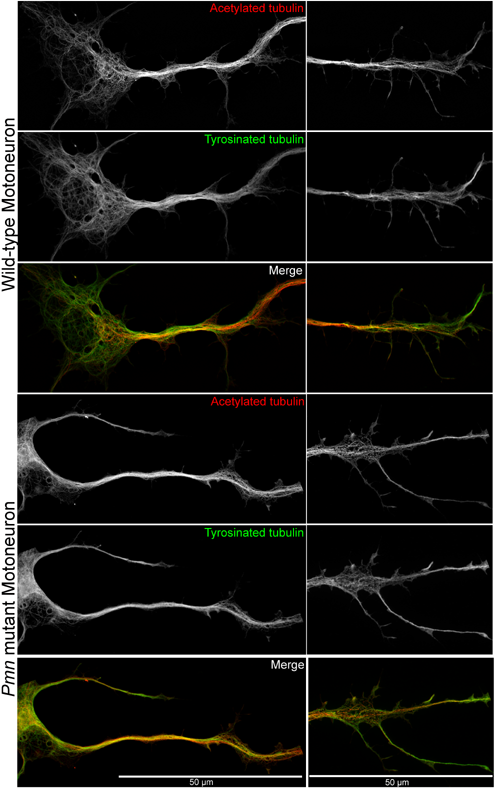

Supplement: Supplementary file 3 — Fig. S3 Distribution of acetylated and tyrosinated tubulin in axons of wild-type and pmn motoneurons as revealed by high resolution SIM. Motoneurons were cultured for 3 days in vitro. Representative images of proximal (left) and distal axon (right) of wild-type and pmn motoneurons, stained with antibodies against tyrosinated α-tubulin (green), acetylated α-tubulin (red). Scale bar 50 µm. (TIFF 254 kb) [file 401_2016_1564_MOESM3_ESM.tif]

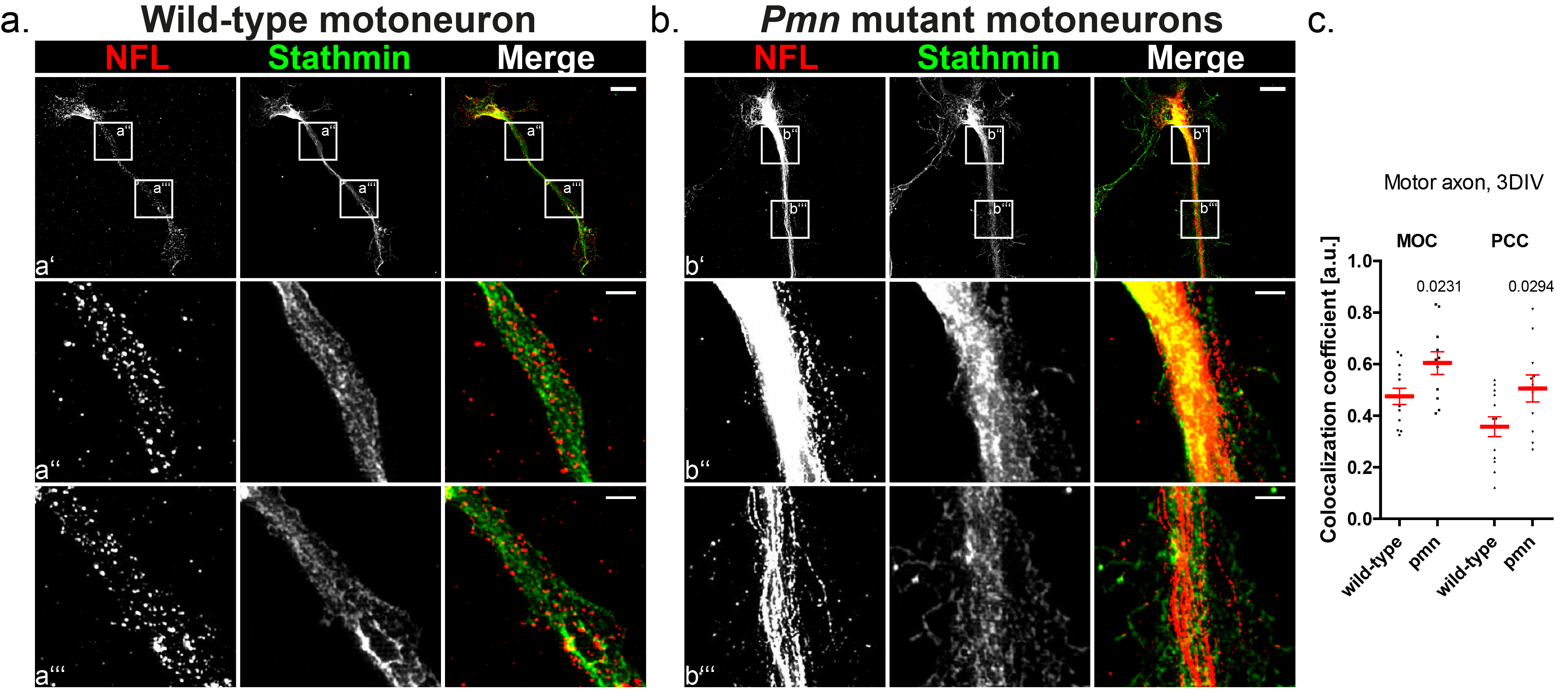

Supplement: Supplementary file 4 — Fig. S4 Subcellular localization of NFL and stathmin in wild-type (a) and pmn (b) motoneurons by high resolution SIM (upper panels, scale bar: 10 µm). Motoneurons were cultured for 3 days in vitro and stained with antibodies against NFL (red, DA2 clone, EnCor Biotechnology) and stathmin (green, rabbit monoclonal, Abcam). White square boxes indicate proximal (‘‘) and distal (‘‘‘) axonal sections which are enlarged in the corresponding lower panels (scale bar: 2 µm). (c) The degree of colocalization between NFL and stathmin appeared increased (MOC: P = 0.0231, t = 2.443, df = 22; PCC: P = 0.0294, t = 2.330, df = 22; unpaired t test) in pmn motor axons (N = 11, MOC = 0.60 ± 0.04, PCC = 0.51 ± 0.05) in comparison to wild-type motor axons (N = 13, MOC = 0.48 ± 0.03, PCC = 0.36 ± 0.04). Quantitative colocalization analysis was carried out in motor axons in a representative culture using the Manders Overlap Coefficient (MOC) and Pearson’s correlation coefficient (PCC) plugins of ImageJ. N is the number of motoneurons analyzed. (TIFF 2621 kb) [file 401_2016_1564_MOESM4_ESM.tif]
